# Assessing hair cortisol levels and cognitive function in older palliative care patients: a potential for broader clinical application of a stress biomarker

**DOI:** 10.1192/j.eurpsy.2025.1743

**Published:** 2025-08-26

**Authors:** R. Ribić, M. Neuberg, T. Košanski, I. Herak, T. Meštrović

**Affiliations:** 1 Department of Nursing, University North, Varaždin, Croatia

## Abstract

**Introduction:**

Older patients in palliative care often experience considerable stress due to physical, emotional and existential factors. Previous research has identified cortisol, a glucocorticoid hormone, as a key biomarker for stress assessment. This pilot study aimed to investigate the potential of hair cortisol as a potentially objective stress biomarker in a specific population (aiming for a broader aplicability), as well as to explore cognitive changes in older palliative patients using the Mini-Mental State Examination (MMSE-2).

**Objectives:**

This study objectives were to (1) assess changes in hair cortisol levels and cognitive function in older palliative care patients over three weeks of hospitalization and (2) evaluate the suitability of hair cortisol as a short-term stress biomarker in this patient group.

**Methods:**

This monocentric pilot study included 19 patients from different palliative care hospital services in Croatia, gathered via a convenience sampling approach with strict inclusion/exclusion criteria. Hair cortisol levels were measured at baseline and after three weeks using an enzyme-linked immunosorbent assay (ELISA). Cognitive function was assessed using the 16-point MMSE-2. Statistical analyses included paired t-tests and linear regression, and significance was set at p<0.05.

**Results:**

A statistically significant increase in mean hair cortisol levels was observed after three weeks of hospitalization (p=0.007), suggesting heightened stress over time. In contrast, MMSE-2 scores showed no statistically significant change (p=0.064), indicating no detectable cognitive decline within the study period. No significant correlations were found between cortisol levels and MMSE-2 scores at either time point, and differences between male and female patients were not statistically significant.

**Conclusions:**

The findings support the potential use of hair cortisol as a biomarker for stress in palliative care settings, especially for tracking the transition from acute to chronic stress. However, MMSE-2 may not be sensitive enough to detect cognitive changes over short time spans in this patient group. Consequently, further research with larger samples is needed to validate hair cortisol as a practical tool for monitoring stress and to explore its clinical implications for improving palliative care outcomes.

**Disclosure of Interest:**

None Declared

